# A Novel Adjustable EndoButton Fixation Assisted by 3D Printing Technology for Tibiofibular Syndesmosis Injury: A Biomechanical Study

**DOI:** 10.3389/fbioe.2022.793866

**Published:** 2022-03-10

**Authors:** Lei Zhang, Junjie Xu, Xiangyu Tang, Xin Zhou, Bingkun Li, Guoyou Wang

**Affiliations:** ^1^ Department of Orthopedics, The Affiliated Traditional Chinese Medicine Hospital of Southwest Medical University, Luzhou, China; ^2^ Center for Orthopedic Diseases Research, The Affiliated Traditional Chinese Medicine Hospital of Southwest Medical University, Luzhou, China; ^3^ Specialist Workstation in Luzhou, Luzhou, China; ^4^ Clinical Base of The Affiliated Traditional Chinese Medicine Hospital of Southwest Medical University, Guangdong Province Medical 3D Printing Application Transformation Engineering Technology Research Center, Luzhou, China; ^5^ School of Clinical Medicine, Southwest Medical University, Luzhou, China

**Keywords:** 3D printing, navigation template, tibiofibular syndesmosis, biomechanics, EndoButton

## Abstract

**Purpose:** The recommendations for surgical fixation of tibiofibular syndesmosis injuries are increasingly challenging for many clinical orthopedists, as international consensus has not been published for the optimal treatment of the injury. Thus, we have created a 3D-printed navigation template for a precise bone tunnel and a novel adjustable EndoButton fixation (NAE) for the ideal treatment. The purpose of this research was to evaluate the accuracy of the 3D-printed navigation template and explore the biomechanical performance of the NAE technique by comparing it with the intact syndesmosis, screw technique, and TightRope (TR) technique.

**Methods:** Twenty-four human cadaveric legs were randomly allocated to four groups: the NAE group (*n* = 6), TR group (*n* = 6), screw group (*n* = 6), and intact group (*n* = 6). A personalized navigation template based on computed tomography scans was designed, and 3D printing models were generated for the distal tibiofibular syndesmosis. The NAE, TR, and screw group were performed via 3D-printed navigation template, respectively. All groups were tested under increasing loading forces including axial loading (from 100 N to 700 N) and torsional loading (from 1 N to 5 N), which were performed in different ankle positions. The displacements of the tibiofibular syndesmosis were analyzed using the Bose Electroforce 3510-AT biomechanical testing equipment.

**Results:** Surgical fixations were conducted successfully through a 3D-printed navigation template. Both in axial or torsional loading experiments, no statistically significant difference was observed in the displacements among the NAE, TR, and intact groups in most situations (*p* > 0.05), whereas the screw group demonstrated obviously smaller displacements than the abovementioned three groups (*p* < 0.05).

**Conclusion:** The 3D printing technology application may become beneficial and favorable for locating and making the bone tunnel. Also, the NAE fixation provides the performance of complete ligaments; it also restores physiologic micromotion and avoids insufficient or excessive reduction when compared to the TR and screw technique. This may offer a new fixation for the treatment of tibiofibular syndesmosis injuries that is desirable for clinical promotion.

## Introduction

The separation of the distal tibiofibular syndesmosis is a common orthopaedic injury that is usually associated with 1–20% of all ankle sprains and 13% of ankle fractures in patients (Egol et al., 2010; Liu et al., 2018; Shimozono et al., 2019; Corte-Real and Caetano, 2021). Inappropriate treatment of syndesmosis injuries could result in ankle instability, stiffness, and poor functional performance and ultimately lead to traumatic osteoarthritis (Krähenbühl et al., 2019). Therefore, it is necessary to search for an ideal treatment when dealing with syndesmotic injuries. For most unstable syndesmosis injuries, with ankle fractures, patients are advised to be treated operatively (van Dijk et al., 2016). Various therapies have been used in clinical practices for years; however, the optimal surgical fixation is still controversial (Teramoto et al., 2011). Although screw fixation is one of the most common methods, it does not respect the dynamic properties, and there still exist some inevitable complications, including screw loosening and breakage and a high risk of reoperation for screw removal (McBryde et al., 1997; Jurkowitsch et al., 2016; Azoulay et al., 2020). More recently, most surgical fixations for syndesmosis diastasis have been operated with the flexible fixation method involving the suture button (Chen et al., 2019; Alastuey-López et al., 2021). Based on suture button design, TightRope (TR) has become a relatively new operation which provides accurate reduction and anatomical maintenance (Qu et al., 2017). However, the TR system fixation also brings about several new potential complications, which include knot infection and looseness (Xie et al., 2018; Pang et al., 2019).

**FIGURE 1:**
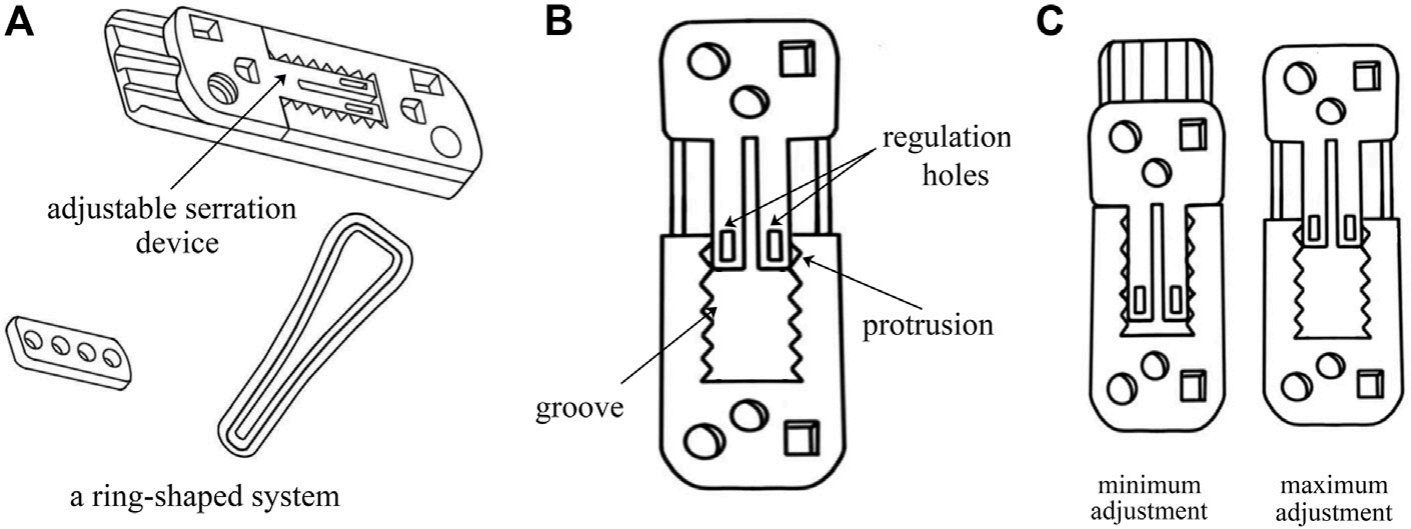
The detail information of the NAE: **(A)** the EndoButton and the suture formed a ring-shaped system; **(B)** the frontal view of the adjustable serration device including regulation holes, special grooves, and protrusions; and **(C)** the minimum and maximum adjustment of the EndoButton.

**FIGURE 2:**
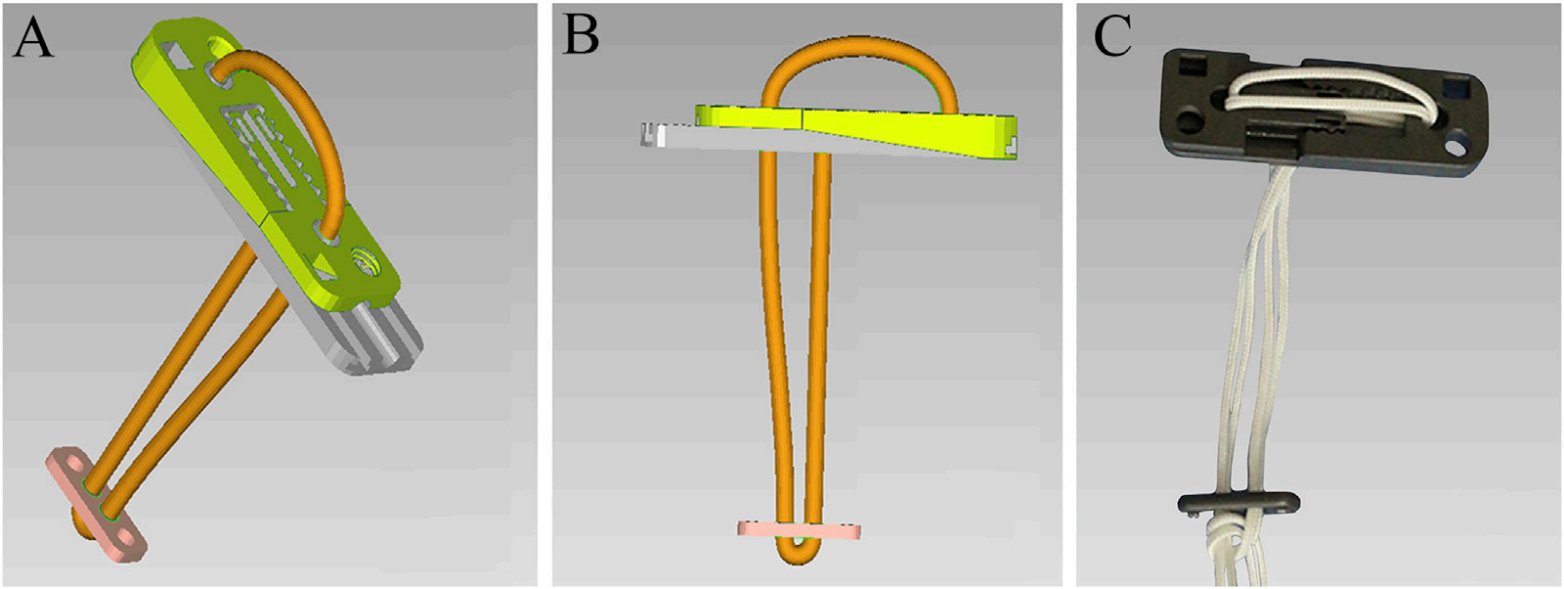
Virtual 3D models of the NAE **(A)** and **(B)** and real product **(C)**.

Consequently, to solve these issues, a novel adjustable EndoButton (NAE) has been introduced with the benefit of being knotless and efficient. In particular, the design behind this innovation is to adjust the length of the loop and the force of reduction intraoperatively according to individual therapy (Weng et al., 2020). In our technique, the EndoButton and the loop formed a ring-shaped system that makes the new fixation stable (Figure 1A). With an adjustable serration device, our EndoButton can change the position of special grooves and protrusions by sliding the regulation holes (Figure 1B), so as to find a suitable force to restore the anatomical relationship of syndesmosis with a controllable range between the minimum and maximum adjustment (Figure 1C). Eventually, the NAE can be adjusted prior to, during, or after fixation of the tibiofibular syndesmosis injury. Virtual 3D models of the NAE and real product are displayed in Figure 2. However, precise placement of the NAE in the distal tibiofibular syndesmosis remains a challenging job that is highly crucial for fixation success. To date, three-dimensional (3D) printing models based on individualized navigation templates have been popular in orthopedic surgery (Jastifer and Gustafson, 2017; Guo et al., 2019; Li et al., 2020). Compared to traditional surgical techniques, the 3D-printed navigation template promotes the accuracy of implant insertion and considers the biomechanical parameters of a specific patient. However, rare is the clinical application in the tibiofibular syndesmosis injury.

Thus, in this study, we designed a 3D-printed navigation template to improve the accuracy of drilling in the tibiofibular syndesmosis during implantation, and cadaveric specimens were used to compare the biomechanical differences between the NAE and two different implants for the treatment of syndesmosis injuries.

## Materials and Methods

### Ethics Statement

This research was approved by the medical ethics committee of The Affiliated Traditional Chinese Medicine Hospital of Southwest Medical University (No. KY2018043).

### Specimens and Grouping

A total of 24 human leg specimens (12 males and 12 females) were included in this study, and the mean age of the specimens was 43.2 years (in the range of 28–62 years). The specimens were chosen from the School of Basic Medical Sciences, Southern Medical University, Guangzhou, China. All of them had been observed carefully by X-ray and CT examinations to confirm their normality. Cadaveric specimens with ankle abnormalities, fractures, ligament lesions, or other serious associated injuries were excluded, and included specimens showed no damage to the tibia, fibula, and ligaments. All cadavers were randomly allocated to four groups: the NAE group (Delta Medical, Beijing, China, *n* = 6), TR group (Arthrex, Naples, FL, *n* = 6), screw group (Delta Medical, Beijing, China, *n* = 6), and intact group (*n* = 6).

### Design and 3D-Printied Navigation Template

**FIGURE 3:**
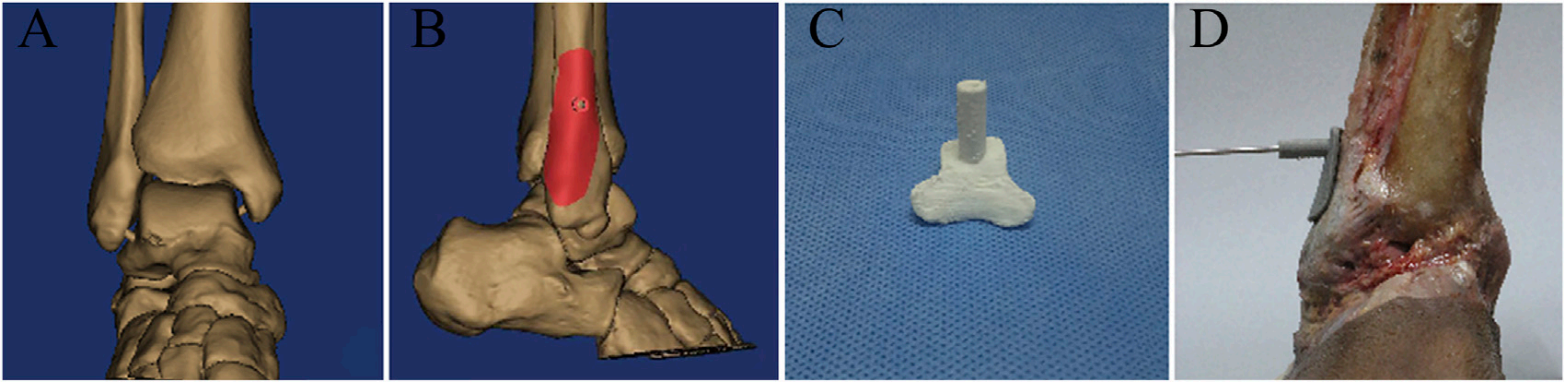
**(A)** 3D model of the ankle joint based on CT reconstruction; **(B)** digital navigation template according to the 3D model; **(C)** 3D-printed navigation template for tibiofibular syndesmosis; and **(D)** the bone tunnel was established with the guidance of a 3D-printed navigation template.

All cadaveric specimens underwent thin-slice CT scanning, and image data were collected from a 64-row spiral CT scan (Siemns, Germany, 120 kV, 120 mAs, 0.6 mm slices, 256 × 256). The DICOM format files of image data were imported into Mimics 21.0 (Materialise, Belgium). In the 3D visualization interface of the Mimics software, the 3D models of the tibiofibular syndesmosis were reconstructed via the Calculate 3D tool and saved as Standard Triangulation Language (STL) format files. Then, the bone plane of the syndesmosis is marked and cut on the 3D model to copy the virtual bone tunnel of fixation surgery. The “Create cylinder” function was used to create a cylinder with the same diameter as the Kirschner wire. By adjusting the length and direction of the virtual Kirschner wire, the position of the bone tunnel was determined along the bone cutting plane. Based on Boolean subtraction, a personalized navigation template was established with holes for drilling guidance. This template design was exported as an STL file and then printed by using a 3D printer (MakerBot Replicator 2, MakerBot Industries, United States) with polylactic acid (PLA). The following process settings were standardized: extruder temperature 215°C, chamber temperature 24°C, primary layer height 0.2 mm, infill 2%, support infill 20%, and maximum overhang without support 60%. Then, a 1: 1 physical model of the template was fabricated with PLA. Finally, we placed the 3D-printed navigation template and fixed it on the cadaveric specimens to assist the syndesmosis drilling (Figure 3).

### Specimen Preparation and Surgical Approaches for Different Fixations

In order to prepare for surgical procedures, the fresh specimens were tested at room temperature and kept wet with normal saline throughout the study. The skin, fascia, muscles, and soft tissues, including the periosteum, were removed carefully, and finally, the tibiofibular syndesmosis was exposed completely. Then, the distal tibiofibular syndesmosis was removed to simulate syndesmosis injury models (except for the intact group).

**FIGURE 4:**
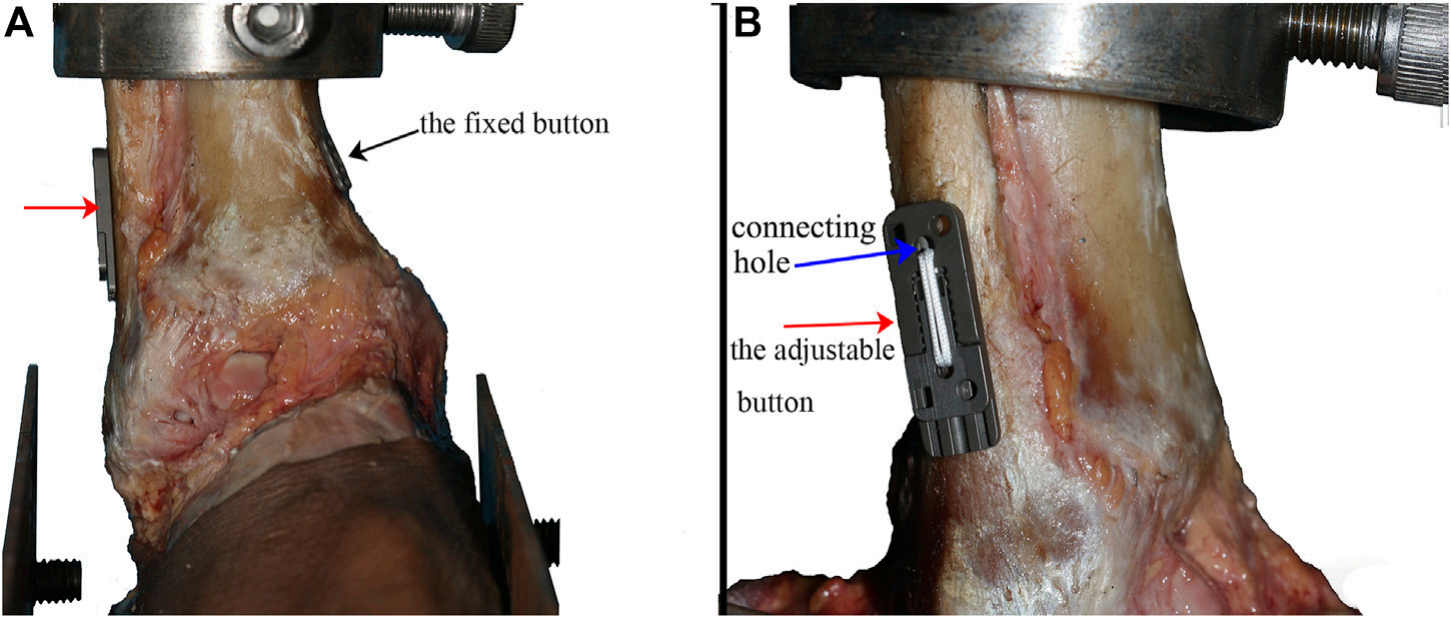
Specimen fixed with the NAE (the black arrow showed the fixed button, the red arrow showed the adjustable button, and the blue arrow showed the connecting holes in the adjustable button) **(A)** observed from the front side and **(B)** observed from the fibula side.

For the NAE group, tibiofibular syndesmosis was secured with the assistance of a patellar clamp in the position of slight dorsiflex (5°). Then, after the 3D-printed navigation template was placed and fixed, a 1.5 mm Kirschner wire was used to make a bone tunnel, drilling from the fibula to the tibia. Then, the template was removed, and the Kirschner wire was placed to assist the knotless loop passing through the bone tunnel. The suture on the fibular side crossed the connecting holes of the adjustable button first, via the bone tunnel. The suture on the tibial side crossed the connecting holes of the fixed button. When finishing the ring-shaped system, the fixed button was pulled on the tibia while the adjustable button was attached to the fibula (as shown in Figure 4). Typically, the length of knotless suture was changed depending on every specimen’s shape by sliding the regulation holes in the adjustable serration device.

For the TR group, this construction consists of two cortical buttons connected by two loops. The surgical method was basically similar to the NAE. The difference, however, was that a knot was needed after fixation to make a circle.

For the screw group, a hole was drilled with the navigation template, and a 3.5 mm screw crossed the hole from the fibular side to the tibia side. Care must be taken to ensure that the screw on each specimen is threaded through the four layers of the tibia and fibula cortex.

### Biomechanical Study

**FIGURE 5:**
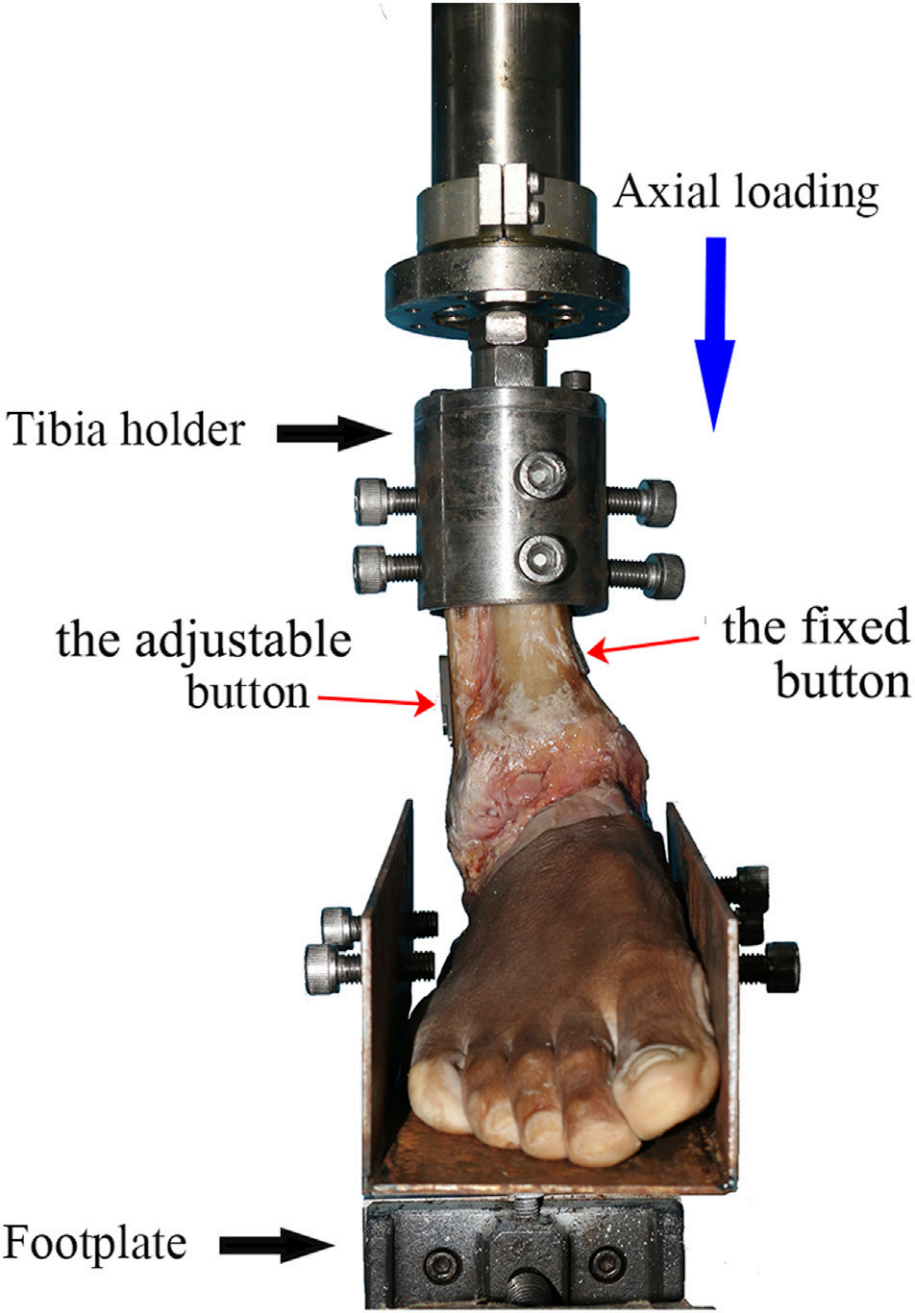
Specimen mounted on the biomechanical testing equipment. Axial and torsional loading experiments were conducted on the equipment. The NAE was inserted into the tibiofibular syndesmosis (shown by the red arrows).

All biomechanical experiments were conducted using the Bose 3510-AT Electro Force biomechanical testing equipment (Bose Corporation, MN, United States) and the Win Test Digital Control System linked to the equipment for transmitting the input and output parameters. This test system had a maximum dynamic load of 7.5 kN, a dynamic displacement of 25 mm, and a test frequency from static to 100 Hz. The cadaveric specimens were rigidly secured to a footplate using a clamping facility, and each ankle was connected to the equipment (as shown in Figure 5). Additionally, the KA-300 grating ruler (Lokshun, Guangzhou, China) was placed on the tibia side and fibula side with an aim of measuring the displacements. The resolution of the KA-300 grating ruler was 1/5 μm.

For the axial loading experiment, the human cadaveric specimens were tested in five different positions: neutral position, plantar flexion (15°), dorsiflexion (10°), valgus (15°), and varus (10°), and the load was exerted along the long axis of the tibia, respectively. Every specimen’s long axis of the tibia must be aligned with the internal/external rotational servomotor, and its transverse bimalleolar axis was supposed to be aligned with the plantar flexion/dorsiflexion axis of the foot plate. The axial loading ranged from 0 to 700 N, and it was applied to all specimens by increasing the speed of 10 N/s steadily by using the biomechanical machine. Before the initial loading, a preloading of 250 N was exerted and slowly pulled out to all the specimens; additionally, ligaments were kept fresh and moist all the time. The data of displacement was acquired for every 20 N increase in the force loading at a frequency of 0.1 Hz, with the load–displacement curve recorded by using the KA-300 grating ruler. Before each load, the limit bar of the KA-300 grating ruler should be adjusted to be close to one end of the syndesmosis. In addition, the data display of the grating ruler was cleared before each load experiment. When reaching the upper limit, the loading was slowly released to the initial value.

For the torsional loading experiment, internal/external rotation torque was applied only in the neutral position. In addition, each specimen was mounted in the same way as in the previous experiment. The axial loading was from 0 to 300 N, and the rotation torque was forced on all specimens from 0 to 5 Nm at a speed of 0.1 Nm/s. Before the initial loading, a 2 Nm preloading force was applied and slowly pulled out to all the specimens. The displacement data were acquired when adding 1 Nto the torsional loading, and the frequency was 0.1 Hz, with the load–displacement curve recorded by using the KA-300 grating ruler.

The abovementioned experiments were supposed to be performed three times repeatedly with an interval of 2 min. The sampling results were the average of three times.

### Statistical Analysis

Quantitative values were presented as mean ± SD. The Anderson–Darling test was used to test the normal distribution, and the Fisher test was used to test the homogeneity of variance. Single-factor analysis of variance (ANOVA) was adopted to detect the difference among the four groups. Data analysis was carried out by the SPSS 20.0 package (SPSS Inc., Chicago, IL, United States), and the level of significance was set at *p* < 0.05.

## Results

### Tibiofibular Syndesmosis Drilling

A total of 18 navigation templates (NAE, TR, and screw groups) were created via 3D printing technology. In each of the abovementioned three groups, the bone tunnel was drilled accurately and successfully via a 3D-printed navigation template, with no need for repeat drilling.

### Displacement in an Axial Loading Experiment

As found in Table 1, in most situations (neutral, dorsiflexion, valgus, and varus, except 300 N in the plantar flexion position), the screw group demonstrated smaller displacements than any of the other three groups of all seven testing points (100–700 N) (*p* < 0.05). Moreover, we found a statistically non-significant difference in the displacements among the NAE, TR, and intact groups at most of these testing points (except 100, 400, 600, and 700 N in the varus position, the TR and NAE groups were smaller than the intact group).

**TABLE 1:** Displacement in different ankle positions.

Fixation	Ankle position	Displacement under different axial loading forces (mm)						
		100 N	200 N	300 N	400 N	500 N	600 N	700 N
Intact	Neutral position	10.504 ± 3.732	11.396 ± 3.842	12.522 ± 4.076	13.063 ± 4.089	13.508 ± 4.094	13.903 ± 4.076	14.157 ± 4.004
	Dorsiflexion	10.012 ± 2.119	10.790 ± 2.186	11.798 ± 2.297	12.260 ± 2.336	12.507 ± 2.328	12.595 ± 2.278	12.893 ± 2.280
	Plantar flexion	10.067 ± 3.083	11.294 ± 3.502	12.209 ± 3.57	13.085 ± 3.804	14.011 ± 4.078	14.027 ± 4.021	15.313 ± 4.440
	Varus	9.304 ± 1.228	10.758 ± 2.228	11.547 ± 2.383	12.625 ± 2.569	12.731 ± 2.623	13.555 ± 2.577	14.157 ± 2.665
	Valgus	9.975 ± 1.535	11.352 ± 1.683	12.112 ± 1.69	12.696 ± 1.701	13.001 ± 1.67	13.408 ± 1.642	13.767 ± 1.614
Screw	Neutral position	4.899 ± 1.561^a^	5.397 ± 1.557^a^	5.807 ± 1.567^a^	6.393 ± 1.591^a^	6.992 ± 1.632^a^	7.768 ± 1.634^a^	8.208 ± 1.655^a^
	Dorsiflexion	4.420 ± 1.374^a^	5.175 ± 1.580^a^	5.889 ± 1.564^a^	6.265 ± 1.648^a^	6.710 ± 1.738^a^	7.385 ± 1.820^a^	7.951 ± 2.100^a^
	Plantar flexion	4.617 ± 1.584^a^	5.218 ± 1.728^a^	5.942 ± 1.910	6.596 ± 1.881^a^	7.158 ± 1.926^a^	7.744 ± 2.107^a^	8.402 ± 2.050^a^
	Varus	5.131 ± 1.471^a^	5.611 ± 1.499^a^	5.963 ± 1.502^a^	6.420 ± 1.494^a^	6.714 ± 1.440^a^	7.159 ± 1.410^a^	7.621 ± 1.477^a^
	Valgus	5.037 ± 1.966^a^	5.258 ± 1.867^a^	5.760 ± 1.966^a^	6.150 ± 1.936^a^	6.450 ± 1.906^a^	6.600 ± 1.790^a^	6.928 ± 1.749^a^
Novel EndoButton	Neutral position	8.592 ± 2.050^b^	9.654 ± 1.918^b^	10.302 ± 2.000^b^	10.851 ± 2.061^b^	11.287 ± 2.092^b^	11.682 ± 2.117^b^	12.005 ± 2.104^b^
	Dorsiflexion	8.164 ± 2.531^b^	9.107 ± 3.154^b^	9.804 ± 3.399^b^	10.378 ± 3.475^b^	10.847 ± 3.510^b^	11.256 ± 3.538^b^	11.610 ± 3.561^b^
	Plantar flexion	7.785 ± 1.285^b^	8.818 ± 1.159^$^	9.653 ± 1.198	10.221 ± 1.321^$^	10.736 ± 1.520^a^ ^$^	11.173 ± 1.714^$^	12.706 ± 2.093^$^
	Varus	7.126 ± 0.831^a^ ^b^	8.813 ± 1.788^b^	9.561 ± 1.820^b^	10.136 ± 1.827^a^ ^b^	10.646 ± 1.850^b^	11.105 ± 1.865^a^ ^b^	11.53 ± 1.915^a^ ^b^
	Valgus	7.571 ± 3.602	8.358 ± 3.878	8.929 ± 3.989	9.402 ± 4.044	9.866 ± 4.099	10.235 ± 4.096	10.609 ± 4.095
Tightrope	Neutral position	8.524 ± 3.019^b^	9.672 ± 3.321^b^	10.353 ± 3.452^b^	10.889 ± 3.493^b^	11.369 ± 3.567^b^	11.474 ± 3.578^b^	11.913 ± 3.648^b^
	Dorsiflexion	7.778 ± 1.995^b^	8.870 ± 1.368^b^	9.749 ± 1.039^b^	10.629 ± 0.928^b^	11.225 ± 0.732^b^	11.722 ± 0.617^b^	12.332 ± 0.660^b^
	Plantar flexion	8.744 ± 2.006^b^	9.498 ± 1.864^b^	10.703 ± 1.863	11.475 ± 1.782^b^	11.933 ± 1.624^b^	12.622 ± 1.794^b^	13.626 ± 2.883^b^
	Varus	5.705 ± 1.692^a^	6.342 ± 3.088	8.85 ± 1.471^a^ ^b^	9.786 ± 1.573^a^ ^b^	10.442 ± 1.592^a^ ^b^	11.021 ± 1.538^a^ ^b^	11.611 ± 1.517^a^ ^b^
	Valgus	9.121 ± 2.660^b^	10.450 ± 3.086^a^	11.455 ± 3.499^b^	12.322 ± 3.781^b^	12.575 ± 3.922^b^	13.005 ± 4.022^b^	13.848 ± 4.213^b^

a Significant difference compared with the intact group (*p* < 0.05).

b Significant difference compared with the screw group (*p* < 0.05).

### Displacement in the Torsional Loading Experiment

As found in Table 2, in the internal and external rotation, the screw group was smaller than the NAE, TR, and intact groups for all five testing points in the displacement comparison (1–5 Nm) (except for 2 Nm in the external rotation) (*p* < 0.05). In addition, the comparison among the NAE, TR, and intact groups was represented without a statistically significant difference of all five testing points (1–5 Nm) (except for 1 Nm in the internal rotation) (*p* < 0.05).

**TABLE 2:** Displacement in internal and external rotation.

Fixation	Rotation	Displacement under different torques (mm)				
		1 N	2 N	3 N	4 N	5 N
Intact	Internal	6.227 ± 0.943	6.280 ± 0.940	6.316 ± 0.936	6.414 ± 0.905	6.635 ± 0.798
	External	8.195 ± 0.484	8.255 ± 0.477	8.280 ± 0.544	8.346 ± 0.537	8.392 ± 0.513
Screw	Internal	3.376 ± 0.395^a^	3.426 ± 0.400^a^	3.498 ± 0.382^a^	3.563 ± 0.380^a^	3.636 ± 0.420^a^
	External	5.351 ± 0.384^a^	5.401 ± 0.393^a^	5.455 ± 0.391^a^	5.507 ± 0.389^a^	5.574 ± 0.396^a^
Novel EndoButton	Internal	5.330 ± 0.923^a^ ^b^	6.440 ± 0.422^b^	6.584 ± 0.349^b^	6.717 ± 0.313^b^	6.780 ± 0.302^b^
	External	7.033 ± 1.229^b^	7.342 ± 1.208^b^	7.472 ± 1.175^b^	7.668 ± 1.193^b^	7.864 ± 1.238^b^
Tightrope	Internal	4.442 ± 0.116^a^ ^b^	4.451 ± 0.114^a^ ^b^	4.478 ± 0.118^a^ ^b^	4.524 ± 0.161^a^ ^b^	4.564 ± 0.198^a^ ^b^
	External	7.152 ± 1.456^b^	7.229 ± 1.490	7.317 ± 1.484^b^	7.402 ± 1.507^b^	7.559 ± 1.561^b^

a Significant difference compared with the intact group (*p* < 0.05).

b Significant difference compared with the screw group (*p* < 0.05).

## Discussion

The major advantages of syndesmosis fixation with the NAE technique are achieving a flexible system, adjusting the length of the knotless loop and the force of reduction, and decreasing procedure complications as far as possible. Furthermore, the improved NAE technique has allowed for accurate drilling with the assistance of 3D printing technology. With the increasing availability of 3D printing, it is becoming popular to assist the surgeon in orthopedic surgery by creating navigation templates and 3D models (Deng et al., 2016). In traditional surgery, limited surgical view makes it challenging to establish a precise bone tunnel during the drilling procedure. It is unknown whether the 3D-printed navigation template may promote the accuracy of drilling in the clinical application of the syndesmosis injury so far. In this study, the personalized 3D-printed navigation template was designed and applied. Finally, the results have shown that the real bone tunnel was accurate, and syndesmosis fixations were conducted successfully along with the guidance of the special navigation templates in the NAE, TR, and screw groups. Apparently, the feasibility of adopting a 3D-printed navigation template in syndesmosis fixation requires evaluation and exploration before clinical applications. Nevertheless, the long-term clinical effects and procedure complications need to be explored in further studies (Xie et al., 2014).

This study investigated the stability and flexibility of the tibiofibular syndesmosis using three different fixation techniques in cadaveric specimens. The results showed that, in axial and torsional loading experiments, the screw group revealed smaller displacements than any of the other three groups in most conditions. This obviously indicated that screw fixation constrained the physiological motion of the tibiofibular syndesmosis compared with the intact model, and the screw technique became too rigid, providing excessive fixation strength. Owing to a rigid system, Kaiser et al. (2021) suggested that screw fixation may be a good option in cases of severe or multiple syndesmotic injuries with unstable fracture comminution. Moreover, technical considerations about screw fixation still remain without clear standards for the location, diameter, orientation, and number of screws inserted (Peek et al., 2014). Surprisingly, among the NAE, TR, and intact groups, a non-significant difference was found statistically in most situations, which meant that these two fixations achieved similar dynamic stability as the intact syndesmosis. Because of the suture button design, NAE and TR allow fibular movement relative to the tibia in the physiological range. Westermann et al. (2014) found that flexible fixation reduced the risk of malreduction and achieved a self-reduction in restoring the anatomical relationship of the distal tibia-fibula. Many studies have reported that flexible fixation is superior to screw fixation when it comes to clinical efficacy and overall postoperative complications (Doll et al., 2020; Gan et al., 2020). However, there are still some shortcomings in the TR technique. For instance, a knot is needed in traditional TR fixation, but because of the knot, suture loosening and knot irritation occur sometimes, making the fixation unstable. While the suture is knotted, the length of the suture cannot be adjusted, and finally, it may cause insufficient or excessive reduction, leading to looseness and hardware removal (Fantry et al., 2017). Naqvi et al. presented a removal rate of 22% in a 2.5-year follow-up study. Also, in a 5-year retrospective case, five patients needed hardware removal owing to persistent knot irritation in 19 patients (26%). In other words, the TR fixation is not perfect and personalized (Naqvi et al., 2012; Förschner et al., 2017).

Accordingly, the NAE is with the goal of retaining the advantages of the TR and screw and improving the disadvantages of them. According to the findings in the study, the NAE fixation might serve the same purpose as TR fixation and complete ligament effect, which means it could provide physiologic stabilization of the ankle joint. With a ring-shaped system, there are no risks of screw removal, loosening, and knot irritation. The second operation for fixation removal is unnecessary (Wei et al., 2021). More importantly, the length of the suture is changeable because of the adjustable serration device, enabling a personalized project according to every patient’s requirements. With a simple construction, it is easy for surgeons to master our novel technique, which can shorten operation time and improve efficiency (Wang et al., 2018).

The current study also has several limitations. First, one limitation was that the study was unable to simulate the effects of living muscle and soft tissue on ankle joint stability because of cadaver specimens. Then, the implant failure tests were not performed in the experiments, so the maximum strengths of the three devices were unknown. Furthermore, large clinical trials are needed to prove the effectiveness of the NAE method. In addition, both the suture and tissues are likely to undergo creep behavior, which means that the measured gap could grow as time passes. Finally, there are frictional forces which could potentially result in suture wear and tearing. For these reasons, further tests could be performed in the future in order to take into account both creep and fatigue behavior, taking advantage of the experimental setup introduced here where sutures are tested on site; therefore, it is possible to best reproduce the actual suture behavior.

In general, the NAE is an effective method for distal tibiofibular syndesmosis injury. This new technique offers physiologic stabilization of the syndesmosis and retains stability and flexibility, which is the same as complete ligaments. In particular, the adjustable length of the knotless loop makes it possible for individualized therapy, and clearly, without a knot, the NAE will decrease the risk of suture loosening and knot irritation. This may offer a new fixation for the treatment of tibiofibular syndesmosis injuries that is desirable for clinical promotion.

## Data Availability

The original contributions presented in this study are included in the article/Supplementary Material, further inquiries can be directed to the corresponding author.
